# Bone marrow lesions related to bone marrow edema syndromes and osteonecrosis

**DOI:** 10.1007/s00132-025-04640-9

**Published:** 2025-04-22

**Authors:** Gad Shabshin, Nogah Shabshin

**Affiliations:** 1 Faculty of Medicine, Tel Aviv University, 35 Klachkin st., Tel Aviv, Israel 6997801; 2 Department of Radiology, Emek Medical Center, Clalit Health Services, Afula, Israel; 3 Department of Radiology, Penn Medicine, Philadelphia, PA USA; 4 16 Ha’Alon st, Raanana, Israel

**Keywords:** Magnetic resonance imaging, Avascular necrosis, Dual-energy computed tomography, Subchondral insufficiency fracture of the knee, Sensitivity and specificity, Magnetresonanztomographie, Avaskuläre Nekrose, Dual-Energy-Computertomographie, Subchondrale Insuffizienzfraktur des Knies, Sensitivität und Spezifität

## Abstract

Bone marrow lesions (BML) are abnormalities in the bone marrow identified on magnetic resonance imaging (MRI) and can generally be classified as traumatic or atraumatic. This review focuses on atraumatic bone marrow edema syndromes (BMES) and their imaging evaluation. The MRI remains the modality of choice for assessing BMES, particularly using fluid-sensitive sequences although other sequences such as Dixon and T1-weighted imaging can be of further assistance. Emerging evidence supports dual-energy CT (DECT) as a reliable alternative, with high sensitivity and specificity for detecting bone marrow edema. The term BMES is a collective term for conditions, such as transient osteoporosis (TO) and regional migratory osteoporosis (RMO), predominantly affect weight-bearing bones in middle-aged individuals and pregnant or postpartum females. Subchondral insufficiency fractures of the knee (SIFK) are a key subset of BMES. These fractures most commonly involve the medial femoral condyle (MFC) and are associated with risk factors, such as meniscal root tears and extrusion of the meniscal body. The MRI findings typically include bone marrow edema-like signals and subchondral fracture lines, with additional features, such as secondary osteonecrosis in advanced cases. Prognostic indicators are crucial for stratifying patients and guiding management. Low-grade or reversible lesions often resolve with conservative treatment, whereas high-grade or irreversible lesions may require surgical intervention.

Avascular necrosis, another atraumatic BML entity, differs from BMES by its association with systemic factors, such as steroid use or alcohol abuse. Accurate imaging, particularly in the early stages, is vital to distinguish between reversible and irreversible lesions, facilitating timely and appropriate management.

## Abstract (de)

Knochenmarkläsionen (BML) sind Anomalien im Knochenmark, die in der Magnetresonanztomographie (MRT) festgestellt und generell als traumatisch oder atraumatisch klassifiziert werden können. Diese Übersicht konzentriert sich auf atraumatische Knochenmarködemsyndrome (BMES) und deren bildgebende Beurteilung. Die MRT ist nach wie vor das Mittel der Wahl für die Beurteilung von BMES, insbesondere unter Verwendung flüssigkeitsempfindlicher Sequenzen, wobei auch andere Sequenzen wie die Dixon- und T1-gewichtete Bildgebung von Nutzen sein können. Neue Erkenntnisse unterstützen die Dual-Energy-CT (DECT) als zuverlässige Alternative mit hoher Sensitivität und Spezifität für den Nachweis eines Knochenmarködems. Der Begriff BMES ist ein Sammelbegriff für Erkrankungen wie transiente Osteoporose und regionale Migrationsosteoporose, die vor allem gewichtstragende Knochen bei Personen mittleren Alters und schwangeren Frauen oder Frauen nach der Geburt betreffen. Subchondrale Insuffizienzfrakturen des Knies (SIFK) sind eine wichtige Untergruppe der BMES. Diese Frakturen betreffen am häufigsten den medialen Femurkondylus (MFC) und sind mit Risikofaktoren wie Meniskuswurzelrissen und Extrusion des Meniskuskörpers verbunden. Zu den MRT-Befunden gehören typischerweise Knochenmarködem-ähnliche Signale und subchondrale Bruchlinien sowie zusätzliche Merkmale wie sekundäre Osteonekrosen in fortgeschrittenen Fällen. Prognostische Indikatoren sind für die Stratifizierung der Patienten und die Steuerung der Behandlung von entscheidender Bedeutung. Geringgradige oder reversible Läsionen lassen sich häufig mit einer konservativen Behandlung beheben, während hochgradige oder irreversible Läsionen einen chirurgischen Eingriff erfordern können. Die avaskuläre Nekrose, eine weitere atraumatische BML-Entität, unterscheidet sich von der BMES durch ihren Zusammenhang mit systemischen Faktoren wie Steroidkonsum oder Alkoholmissbrauch. Eine genaue Bildgebung, insbesondere in den frühen Stadien, ist entscheidend für die Unterscheidung zwischen reversiblen und irreversiblen Läsionen und erleichtert eine rechtzeitige und angemessene Behandlung.

## Introduction

The term bone marrow lesions (BML) refers to abnormalities in the bone marrow that are seen on magnetic resonance imaging (MRI). The BMLs can be classified as traumatic and atraumatic. Atraumatic BMLs can further be classified based on the etiology. These include lesions related to bone marrow replacement or infiltration in conditions such as neoplasms and infections, those related to degenerative processes (e.g., osteoarthritis), bone marrow edema syndromes (BMES) and avascular necrosis (AVN). This review article aims to provide a practical approach to imaging techniques and features of atraumatic subchondral BMES.

## Imaging techniques in bone marrow evaluation

### Magnetic resonance imaging

The MRI is considered the modality of choice to evaluate the bone marrow. Fluid-sensitive sequence is a term that describes sequences in which the fat signal is suppressed, while fluid demonstrates a bright signal. The entire picture is relatively black, structures containing fluid, such as the urinary bladder, cerebrospinal fluid (CSF) and joint fluid appear white but edema and most of the abnormalities appear bright. These sequences include T2 or intermediate weighted imaging (WI) with fat suppression and short tau inversion recovery (STIR). Although these sequences are the most sensitive to demonstrate an abnormality in the bone marrow, they are not specific. High intensity on fluid-sensitive sequences is a mutual characteristic of most BMLs.

T1WI is less sensitive than fluid-sensitive sequences but is more specific

The use of T1WI is less sensitive than fluid-sensitive sequences but is more specific. The T1WI can aid in differentiating between replacement processes (tumors and osteomyelitis) and other types of benign BML. As long as the T1 signal is higher than that of a muscle or intervertebral disc, there is no concern of bone marrow replacement and the suggested terminology by the Society of Skeletal Radiology is bone marrow edema-like signal [10, 25]. If the T1WI signal is isointense or hypointense compared to that of a muscle or disc, it raises a concern of marrow replacement. In such cases, other sequences including Dixon in-phase and out-of-phase imaging can aid in differentiating benign from malignant lesions and osteomyelitis.

**Fig. 1:**
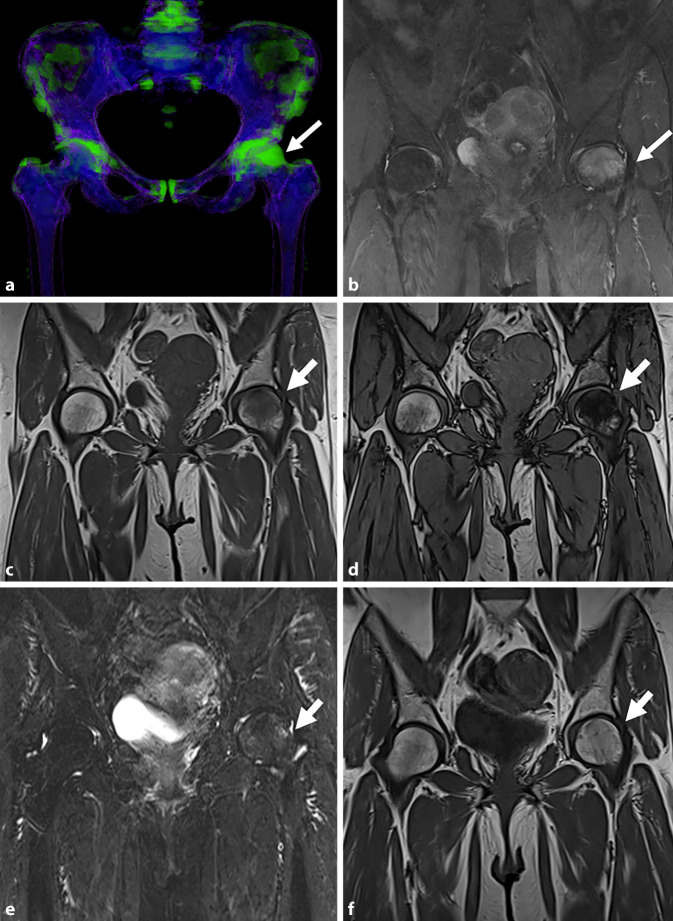
A 55-year-old patient with left hip pain related to reversible left hip bone marrow edema (BME) syndrome. **a** Frontal view dual-energy computed tomography with bone marrow algorithm shows bone marrow edema of the left femoral head (colored green, *white arrow*). **b** Fluid-sensitive magnetic resonance sequence performed 2 weeks later confirmed the bone marrow edema (*white arrow*). **c** T1-weighted Dixon in-phase image shows low signal, isointense to the adjacent muscles (*white arrow*). **d** The out of phase image shows a drop of signal of > 20%, indicating that there is no marrow replacement and that this is a benign lesion (*white arrow*). **e** Coronal fluid sensitive magnetic resonance image performed after 5 weeks demonstrates near complete resolution of the BME (*white arrow*). **f** Coronal T1WI after 5 weeks: the shape of the articular surface is maintained (*white arrow*)

The Dixon sequence, first introduced by Dixon in 1984, is an MRI sequence that has rapidly emerged in recent years [7]. This sequence utilizes one acquisition to produce separation of water and fat signals by creating two images with different phase shifts resulting in three different series: in-phase, opposed phase (out-of-phase) and fat suppression. In 2005, Zajick et al. suggested that more than 20% drop of signal on opposed phase compared to in-phase, is suggestive of a benign lesion while drop of signal of less than 20% is concerning for malignancy [28]. Recently, a study published by Sasiponganan et al. showed that on T2-weighted Dixon sequences the same cut-off values are applicable ([22]; Fig. 1).

### Dual-energy CT

In recent years, another modality, dual-energy computed tomography (DECT) has been used more frequently.

DECT demonstrates high diagnostic accuracy in detecting bone marrow edema in atraumatic patients

The technology was first studied by Alvarez and Macovski in 1976, who laid the foundation for modern dual-energy imaging [2]. When using DECT two different X‑ray energy spectra are utilized to differentiate materials. Images are acquired at two distinct energy levels, typically through methods such as rapid voltage switching of the X‑ray tube, dual-source CT or dual-layer detectors. Musculoskeletal applications of DECT include depiction of bone marrow edema (Fig. 1) on CT and depiction of crystal deposition in gout.

A recent meta-analysis conducted by Chen et al. demonstrated the high diagnostic accuracy of DECT in detecting bone marrow edema in atraumatic patients, reporting a sensitivity of 88.4%, a specificity of 96.1% and an overall accuracy of 98% [4]. This meta-analysis highlighted an even higher diagnostic performance of DECT for bones other than the pelvis, with a specificity of 97%. The robust methodological approach in this article, including subgroup analyses and a low risk of bias across included studies, strengthens the reliability of these findings and supports DECT as an effective alternative to MRI for detection of bone marrow edema.

## Bone marrow edema syndromes

The BMES are a basket of conditions that include transient osteoporosis (TO), regional migratory osteoporosis (RMO) and insufficiency fractures. Some authors also include complex regional pain syndrome (CRPS), also known as algodystrophy.

In recent years, there is an understanding that these pathologies represent different features of the same condition, due to similar imaging features and demographic characteristics. They affect weight-bearing bones, most commonly the knee and hip joints, less frequently the metatarsal head and rarely other bones of the foot and ankle [13]. In most cases BMES affect middle-aged men and women, pregnant and postpartum females [27].

It is well recognized that the subchondral plate provides support to the cartilage

On MRI all BMES demonstrate an extensive subchondral bone marrow edema-like signal with ill-defined margins. The edema can also be seen on DECT (Fig. 1). The edema can be so extensive that in BMES of the proximal femur it can extend to the base of the femoral neck, in BMES of the femoral condyles of the knee it can cross the intercondylar fossa and in the metatarsal head it can extend proximally along the shaft. In most cases the edema resolves, at least partially, over a period of 8 weeks. In some cases, the edema can complicate with subchondral fractures and some of these fractures are associated with osteonecrosis of the subchondral fragment.

The subchondral bone is a component of the osteochondral unit which plays a substantial role in joint health [9]. It is well recognized that the subchondral plate provides support to the cartilage. Subchondral bone injury can lead to loss of articular cartilage possibly because the injury is followed by callus formation that is stiffer than normal subchondral bone. This leads to increased compressive mechanical forces on the cartilage [6]. This explains why some of the BMES, especially those that are complicated with a fracture and secondary osteonecrosis develop osteoarthritis and even more so, rapidly progressive osteoarthritis [8].

### Terminology of BMES

Historically, the term TO of the hip was used before the MRI era to describe osteopenia of the affected bone, when compared to its counterpart in the contralateral side. Later, MRI correlation showed bone marrow edema-like signal in those osteopenic bones [14]. When these imaging features are seen synchronously or metachronously in other subchondral bones, it is considered to be RMO [14]. The term CRPS, also known as algodytrophy, was also used to describe conditions that have a similar appearance on MRI [14]. Some of these cases may be complicated by subchondral insufficiency fractures and of these, some may lead to further necrosis of the subchondral fragment.

In a systematic review aiming to map the diversity and frequency of these diagnostic terms, the authors concluded that the terminology for these closely related conditions is inconsistent and lacks standardization [11]. This absence of clear definitions hinders a precise diagnosis, research into disease mechanisms and effective treatment. Therefore, it may be more accurate to use the term BMES and to further describe them as “with” or “without” a subchondral fracture line and whether they are complicated by osteonecrosis.

The BMES can be classified as reversible and irreversible lesions

The BMES can be classified as reversible and irreversible lesions. If bone marrow edema is the only feature on MRI a lesion is considered reversible and the symptoms are usually self-limiting. The presence of osteonecrosis or its complications (e.g., collapse of the articular surface), classifies a lesion as irreversible [16]. When discussing insufficiency fractures an overlap exists: some of these lesions are reversible, while others will progress to irreversible lesions. Most of the literature regarding insufficiency fractures focuses on the knee, thus, this entity will be discussed in the following subsection.

### Spontaneous insufficiency fractures of the knee

Spontaneous insufficiency fractures of the knee (SIFK) typically affect individuals in their 5th and 6th decades of life. These should be clinically suspected when a patient in this age group complains of either a new onset of pain or worsening of a routine pain that persists during rest and at night. The term SIFK was previously called spontaneous osteonecrosis of the knee (SONK) and was believed to affect mainly women at their 6th decade or older, many of those following arthroscopy; however, this term is incorrect as the necrosis is not spontaneous but secondary to a subchondral insufficiency fracture. Furthermore, some studies demonstrated that SIFK is equally distributed among males and females while others still support the female predominance [5, 27]. The association of SIFK with osteoporosis remains unclear; however, advanced lesions are most common in women. The most significant risk factors include posterior meniscal root tears and extrusion of the meniscal body and the most common location is the medial femoral condyle (MFC) (65%) followed by the medial tibial plateau and lastly the lateral compartment [12, 21, 23, 27]. Most cases are unilateral but bilateral and even multiple condyles may be affected almost simultaneously or at different times. When more than one condyle is involved in the same MRI study, usually one condyle will demonstrate a more severe and extensive edema than the other, as one is at the peak while the other is already at the resolution phase.

Several studies investigated prognostic outcomes and found that those associated with poor outcomes include large lesions (> 5 cm^2^, lesion dimensions exceeding 26 mm, or when the maximum width ratio of the lesion in the anteroposterior view exceeds 40% of the affected condyle or involves more than 50% of the transverse dimension), subchondral band of low T2-weighted signal (length > 14 mm or thickness > 4 mm), extent of cartilage loss and meniscal damage, loss of knee range of motion at presentation, baseline arthritis (K‑L grade 4), older age, SIFK of both medial femoral and tibial plateaus, meniscal extrusion, lateral meniscal root tear and varus malalignment [1, 15, 20, 21, 23].

**Fig. 2:**
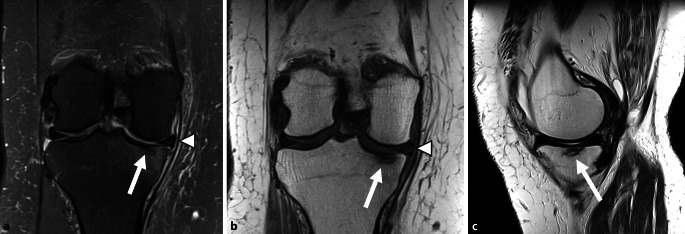
A reversible, low grade subchondral insufficiency fracture of the knee in the medial tibial plateau. **a** A coronal fluid sensitive magnetic resonance image. **b** A coronal T1WI. **c** A sagittal Proton Density weighted imaging. The classical triad of diffuse bone marrow edema, a subchondral fracture line (*arrow*) and medial meniscus body extrusion (*arrowhead*) is noted

**Fig. 3:**
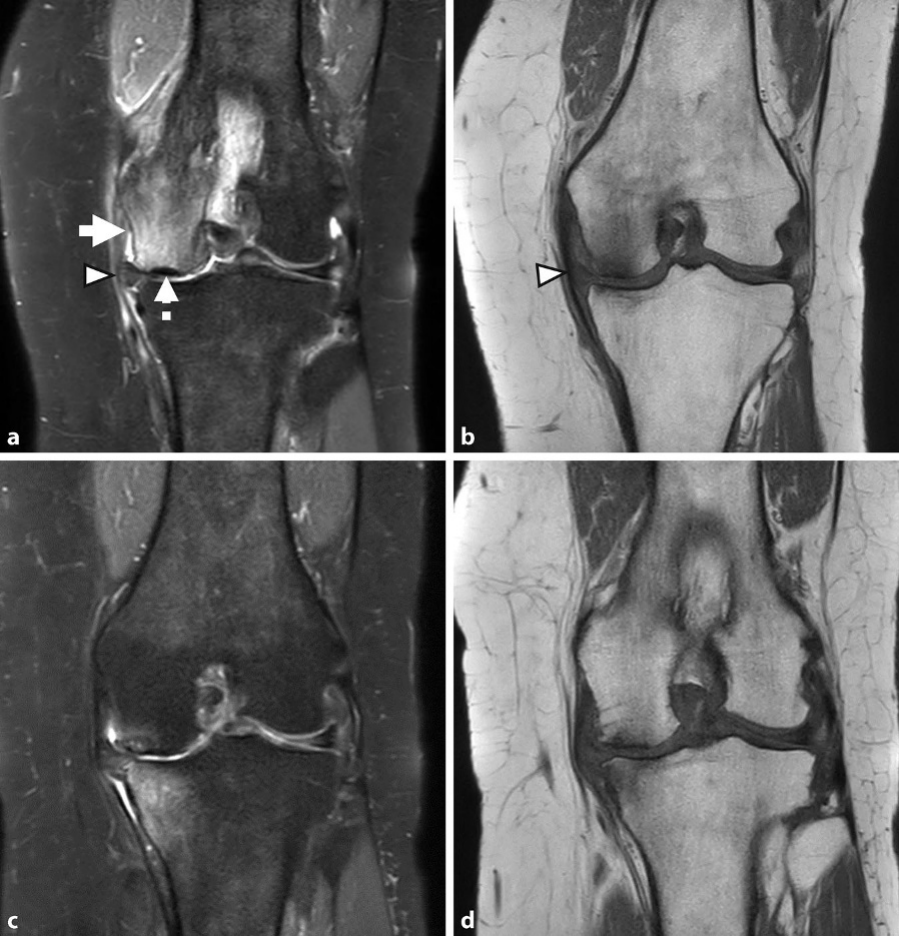
Irreversible, high grade subchondral insufficiency fracture of the knee of the medial femoral condyle (MFC). Coronal Proton Density with fat suppression (**a**) and T1-weighted imaging (**b**): there is diffuse bone marrow edema in the MFC (*arrow*). In addition, there is a band of low signal intensity compatible with osteonecrosis of the subchondral fragment (*dotted arrow* in **a**). Note the severe medial meniscal body extrusion (*arrowhead*). The contour of the articular surface is maintained. There is diffuse cartilage loss. Coronal proton density with fat suppression (**c**) and T1-weighted imaging (**d**) after 1 year. There is advanced osteoarthritis with complete cartilage loss, joint space narrowing and osteophytes

On imaging, in addition to a bone marrow edema-like signal, there is often a thin subchondral fracture line that demonstrates low signal intensity on all sequences and is separated from the articular surface (Fig. 2). Secondary osteonecrosis of the subchondral fracture is seen on MRI as a band of low signal intensity on all sequences (including fluid-sensitive sequences) that is continuous with the articular surface (Fig. 3).

In 2019, Sayyid et al. attempted to establish a grading system to stratify patients suffering from SIFK according to several measurements [23]. This study yielded a new classification of low grade (LG) and high grade (HG) SIFK, in which the latter is associated with worse outcomes. According to this system, surrogate markers of HG lesions include medial meniscal tears at the posterior root attachment with moderate to severe extrusion, HG chondrosis, articular surface collapse and significant perilesional soft tissue edema. In contrast, BME-associated LG SIFK often improves or resolves. In addition, lesion dimensions (anteroposterior > 16.5 mm, transverse > 10.5 mm, mean sum > 26 mm) were shown to help stratify patients with a worse prognosis.

The significance of classifying lesions to LG versus HG or reversible versus irreversible is that while LG/reversible lesions can be treated conservatively, the course of the latter leads to joint replacement [13].

**Fig. 4:**
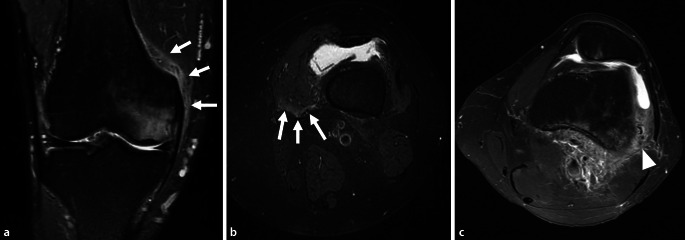
Soft tissue findings associated with subchondral insufficiency fracture of the knee. **a** The metaphyseal burst sign. A coronal fluid sensitive image demonstrates soft tissue edema along the distal femoral metaphysis (*arrows*). **b** Edema is noted along the vastus medialis muscle on an axial fluid sensitive image (*arrows*). **c** A different patient. Characteristic soft tissue edema pattern with extensive edema abutting the posterior femoral cortex, extending along the vastus lateralis fascia is noted on an axial fluid sensitive image (*arrowhead*)

In addition to osseous changes seen on SIFK, typical soft tissue changes have been described. These include edema in the posterior soft tissues of the knee and along the fascia of the vastus medialis and lateralis muscles, edema that surrounds the involved tibia (in tibial plateau SIFK) and the metaphyseal burst sign which describes soft tissue edema along the metaphysis of the involved condyle ([26, 27]; Fig. 4).

#### Avascular necrosis

**Fig. 5:**
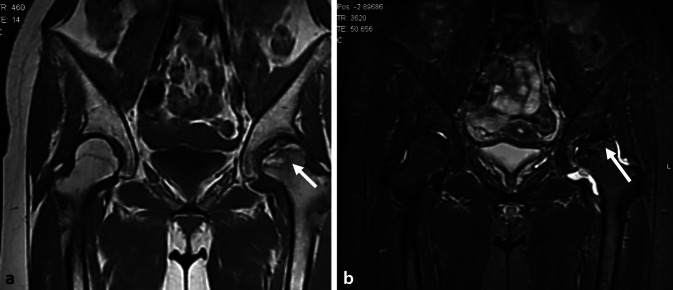
Chronic avascular necrosis (AVN) of the left femoral head. Coronal T1-weighted (**a**) and fluid-sensitive sequence (**b**) frontal views. There is a serpentine line representing the border of the AVN (*arrows*). There is fatty replacement of the necrotized part. The articular surface has collapsed. Osteoarthritis with cartilage loss and joint synovitis are also seen

Avascular necrosis is another type of atraumatic subchondral BML; however, it is a different entity than BMES. Unlike BME syndromes, AVN does not happen spontaneously. It is almost always associated with systemic conditions, such as steroid use, alcohol abuse, sickle cell disease or secondary to a subcapital proximal femoral fracture and is a result of insufficient blood supply [3]. Because it is associated with underlying conditions, there is no association with age, gender or internal derangements of the joint involved. Although less frequently, AVN can also occur in non-weight-bearing bones, such as the humeral head, while BMES typically involve weight-bearing bones and are associated with middle age, internal derangements of the knee and pregnancy. On MRI, acute AVN demonstrates the typical serpiginous double line sign and surrounding BME [17]. During the chronic phase the extensive bone marrow edema greatly improves and even disappears and the necrotized area is replaced by fat and sclerosis ([24]; Fig. 5).

The necrotic volume of AVN has been shown to be predictive of future articular collapse

The necrotic volume of AVN has been shown to be predictive of future articular collapse. A necrotic volume > 30% is a predictor of articular surface collapse, while necrotic volume of < 30% will progress to collapse in less than 5% of the patients [19]. Other factors associated with articular surface collapse include increased joint effusion, bone marrow edema surrounding the AVN, patient age > 40 years and increased body mass index (BMI, ≥ 24 kg/m^2^) [18].

Low-grade lesions can be treated conservatively and high-grade lesions are usually treated surgically

In summary, BMES is a basket of conditions that may vary in clinical and imaging features but have much in common. The use of MRI remains the primary tool for the evaluation of these conditions, especially in the early stages and can provide important information for making decisions. In recent years data supporting the use of DECT have emerged. There are several factors that are associated with poor outcomes of SIFK. Early diagnosis and appropriate management of SIFK and hip insufficiency fractures may improve the prognosis and the disease course of patients. Thus, it is important to know how to differentiate reversible lesions that can be self-limiting, from irreversible lesions that can alter patient management. Low-grade lesions can be treated conservatively and high-grade lesions are usually treated surgically. Future research could aid in further differentiating lesion types and enable a more personalized treatment plan.

## Conclusion

- Bone marrow edema syndromes (BMES) encompass various conditions with overlapping imaging characteristics.
- The primary imaging tool for BMES is MRI, with dual-energy CT emerging as an alternative.
- Early diagnosis and appropriate management are essential for improving patient outcomes.
- Differentiating reversible from irreversible lesions is important as reversible cases can resolve with conservative therapy, while irreversible cases often require surgery.
- Subchondral insufficiency fractures of the knee are a significant subset of BMES, requiring careful assessment to determine the prognosis and treatment.
- Standardized terminology is needed to enhance diagnosis, research and treatment strategies.

## Abbreviations

AVN: Avascular necrosis
BMES: Bone marrow edema syndrome
BMI: Body mass index
BML: Bone marrow lesion
CRPS: Complex regional pain syndrome
CSF: Cerebrospinal fluid
DECT: Dual-energy computed tomography
HG: High grade
LG: Low grade
MFC: Medial femoral condyle
MRI: Magnetic resonance imaging
RMO: Regional migratory osteoporosis
SIFK: Subchondral insufficiency fracture of the knee
SONK: Spontaneous osteonecrosis of the knee
STIR: Short tau inversion recovery
T1WI: T1-weighted imaging
TO: Transient osteoporosis
